# Mast cells in meningiomas and brain inflammation

**DOI:** 10.1186/s12974-015-0388-3

**Published:** 2015-09-17

**Authors:** Stavros Polyzoidis, Triantafyllia Koletsa, Smaro Panagiotidou, Keyoumars Ashkan, Theoharis C. Theoharides

**Affiliations:** Department of Neurosurgery, Kings College London, London, UK; Department of Pathology, AHEPA General Hospital, Thessaloniki, Greece; Molecular Immunopharmacology and Drug Discovery, Department of Integrative Physiology and Pathobiology, Tufts University School of Medicine, 136 Harrison Avenue, Suite J304, Boston, MA 02111 USA; Sackler School of Graduate Biomedical Sciences, Tufts University School of Medicine, Tufts Medical Center, Boston, MA USA; Department of Internal Medicine, Tufts University School of Medicine, Tufts Medical Center, Boston, MA USA; Department of Psychiatry, Tufts University School of Medicine, Tufts Medical Center, Boston, MA USA; Department of Integrative Physiology and Pathobiology, Tufts University School of Medicine, 136 Harrison Avenue, Suite J304, Boston, MA 02111 USA

## Abstract

**Background:**

Research focus in neuro-oncology has shifted in the last decades towards the exploration of tumor infiltration by a variety of immune cells and their products. T cells, macrophages, B cells, and mast cells (MCs) have been identified.

**Methods:**

A systematic review of the literature was conducted by searching PubMed, EMBASE, Google Scholar, and Turning Research into Practice (TRIP) for the presence of MCs in meningiomas using the terms meningioma, inflammation and mast cells.

**Results:**

MCs have been detected in various tumors of the central nervous system (CNS), such as gliomas, including glioblastoma multiforme, hemangioblastomas, and meningiomas as well as metastatic brain tumors. MCs were present in as many as 90 % of all high-grade meningiomas mainly found in the perivascular areas of the tumor. A correlation between peritumoral edema and MCs was found.

**Interpretation:**

Accumulation of MCs in meningiomas could contribute to the aggressiveness of tumors and to brain inflammation that may be involved in the pathogenesis of additional disorders.

## Introduction

Meningiomas are primary extra-axial tumors of the central nervous system (CNS) with an incidence of ~2 in 100,000 in adults [1]. There is an approximate 3:1 female predominance of these tumors mainly attributed to the action of oestrogens [2–4]. While meningiomas represent 15 % of intracranial, 25 % of intraspinal, and 28 % of primary CNS tumors, they usually are not at the center of research focus, probably due to their predominantly benign nature, which results in favorable outcomes. However, a minority of meningiomas is found to be more aggressive, invade brain parenchyma, present with significant degree of peritumoral edema, recur, and subsequently have an aggressive course with increased morbidity and mortality [5].

Meningiomas are commonly associated with headaches, imbalance, vision disturbance, and other neurologic problems [6] that could be quite debilitating. Based on the 2007 (current) classification and grading system of the World Health Organization (WHO) for nervous tissue tumors [7], meningiomas belong to the meningothelial-cell tumors of the meninges and are classified in three grades (Table 1): grade I—slow growing, noncancerous; grade II—atypical with mixed noncancerous and cancerous features; and grade III—cancerous and fast growing. The vast majority of meningiomas is graded as I and is benign [5] with treatment consisting solely of surgical resection, which results in recurrence rates of 2–3 % following total resection [8]. For selected small or incompletely excised tumors, focused radiotherapy is an additional option [9]. However, treatment of meningiomas encasing crucial neural and vascular structures, and of more aggressive histological types, such as anaplastic (grade III), can be more challenging. The current standard of care for the latter comprises surgery and additional radiotherapy, which contributes to a mean overall survival of 3.3 years for anaplastic meningiomas based on a retrospective study [1, 10]. Mean progression-free survival is 11.5 and 2.7 years, respectively [11].

**Table 1:** Classification of meningiomas

1. Grade I. Meningothelial, fibrous (fibroblastic), transitional (mixed), psammomatous, angiomatous, microcystic, secretory, lymphoplasmacyte-rich, metaplastic
2. Grade II. Chordoid, clear cell, atypical, brain-invasive
3. Grade III. Papillary, rhabdoid, and anaplastic (malignant)

Mainstream theory about tumorigenesis of meningiomas favors origin from arachnoid cap cells, while intraventricular ones probably develop from meningothelial inclusion bodies of the tela choroidea arachnoid [12]. Tumor formation can be triggered or affected by various factors, such as genetic [classically the neurofibromatosis 2 (NF2) gene] [13, 14], previous radiotherapy, and possibly head trauma [15, 16]. Meningiomas can be infiltrated by various types of cells, mainly of the immune system, such as macrophages, CD8 lymphocytes, and mast cells (MCs) [17, 18]. Some meningioma variants have been associated with systematic inflammatory syndromes as well, for example, Castleman’s syndrome [19]. This could either represent an innate immune response to the newly forming tumor or could be a factor contributing to tumorigenesis. As such, it has been shown that common variations in the genetics of the innate immune system, such as T-cell regulation, chronic inflammation, IgE, and allergic reactions, may increase the risk of meningioma [20–24]. Presently, the exact nature of this interaction between meningiomas and the immune system, and its potential effect on tumor growth, remains unclear.

A retrospective immunohistochemical study investigated mononuclear cell infiltration in a series of 34 meningiomas [18]. Such infiltrates mainly comprised of T cells and macrophages, with the latter being significantly associated with high cellularity, nuclear atypia, and frequent mitotic figures intra- and perivascularly. MCs were seen in 9/32 (28 %) tumors, among which the most prevalent subtype was the syncytial in agreement with previous studies. Prognostic significance of this finding was unclear in this study.

MCs of various phenotypes were found mainly perivascularly and sporadically in lobules of connective tissue in all types of meningiomas, including malignant ones independent of growth rate, grading, and the degree of calcification [25].

A rare case of a convexity meningioma with an unusually high number of MCs, which presented as chronic subdural hematoma, was described by Popovic et al. [26]. It was suggested that intratumoral histamine release, histamine-associated vasodilation, and subsequent tumoral hemorrhage in MC-rich meningiomas may be an underlying mechanism in a small proportion of meningioma-related subdural hematomas.

Incidental findings on meningiomas and MCs have also been reported in the context of studies with other objectives. During examination of the distribution of the glial fibrillary acidic protein (GFAP) in 131 paraffin-embedded sections of brain neoplasms deriving from either surgical or postmortem specimens [27], MCs were identified in one case of meningothelial meningioma [28].

## Methods

### Objectives

The aim of this study was to review the literature investigating the presence of MCs in meningiomas and their potential interaction. More specifically, the literature was reviewed for the role of this interface and its contribution to tumorigenesis, biological behavior, tumor growth, and tumor suppression.

### Selection of articles

A systematic review of the literature was conducted by a thorough online search in PubMed, EMBASE, Google Scholar, and Turning Research into Practice (TRIP) to retrieve all articles evaluating the presence and role of MCs in intracranial meningiomas. In the PubMed MeSH database system, we used the terms *meningioma, inflammation* and *mast cells [“Meningiomas” (Mesh) AND “Mast cells” (Mesh)]*. Furthermore, to eliminate the chance of missing any other published data, references from past relevant publications were also evaluated. Perspective and retrospective clinical studies, including case reports, as well as experimental studies in animals were included only in the English language.

## Results

### Mast cells in brain inflammation

There is growing interest is the presence of MCs in meningiomas, because of the conflicting reports with regard to the association of MCs with meningioma grade [24, 29, 30]. Furthermore, there is increasing evidence suggesting MCs may stimulate neoplastic growth [31, 32], while others support a potential dual role of MCs contributing both to tumorigenesis and tumor-suppression processes in various types of cancers [33].

MCs originate from a bone marrow progenitor and subsequently develop different phenotype characteristics locally in tissues. Their range of functions is wide and includes participation in allergic reactions, innate and adaptive immunity, inflammation, and autoimmunity [34]. In the human brain, MCs can be located in various areas, such as the pituitary stalk, the pineal gland, the area postrema, the choroid plexus, thalamus, hypothalamus, and the median eminence [35]. In the meninges, they are found within the dural layer in association with vessels and terminals of meningeal nociceptors [36]. MCs have a distinct feature compared to other hematopoietic cells in that they reside in the brain [37]. MCs contain numerous granules and secrete an abundance of prestored mediators such as corticotropin-releasing hormone (CRH), neurotensin (NT), substance P (SP), tryptase, chymase, vasoactive intestinal peptide (VIP), vascular endothelial growth factor (VEGF), TNF, prostaglandins, leukotrienes, and varieties of chemokines and cytokines some of which are known to disrupt the integrity of the blood-brain barrier (BBB) [38–40].

They key role of MCs in inflammation [34] and in the disruption of the BBB [41–43] suggests areas of importance for novel therapy research. Increasing evidence also indicates that MCs participate in neuroinflammation directly [44–46] and through microglia stimulation [47], contributing to the pathogenesis of such conditions such as headaches, [48] autism [49], and chronic fatigue syndrome [50]. In fact, a recent review indicated that peripheral inflammatory stimuli can cause microglia activation [51], thus possibly involving MCs outside the brain.

### Mast cells in meningiomas

MCs have been found to infiltrate both primary and metastatic tumors [52]. For instance, MCs infiltrated and proliferated both in the tumor mass and in the area adjacent to tumor-associated vessels in an experimental model of high-grade gliomas, including glioblastoma multiforme [53]. In addition, stem cell factor (SCF), the main growth factor of MCs, was mainly expressed around the tumor-associated vessels, and it was proposed that the tumor-derived CXCL12/CXCR4 attracted MCs [53].

Polajeva et al. reported that MCs were detected in both low- and high-grade gliomas [54], and it was concluded that (a) MC accumulation in these tumors increased as grade malignancy increased, (b) neutralization of the glioma-derived macrophage migration inhibitory factor (MIF) reduced the extent of MC migration, (c) the magnitude of MC recruitment correlated with the level of MIF, and (d) MIF-induced accumulation of MCs in vivo was associated with activation of the signal transducer and activator of transcription 5 (STAT5) [54]. Additionally, MCs have been detected in increased numbers in the infiltrating zones of medulloblastomas and gliomas [55], while they were also found to be erythropoietin (EPO)-positive in 50 % of a series of hemangioblastoma specimens [56].

Moreover, it has been proposed that metastatic brain tumors can be promoted by stress (unavoidable in patients with cancer) [57] which activates brain MCs to disrupt the BBB via the CRH pathway [58]. This increases BBB permeability for primary cancer cells deriving from the periphery, which can subsequently infiltrate brain parenchyma and metastasize as was shown for rat mammary adenocarcinoma [59]. In two case reports, breast cancer cells were reported to be associated with meningiomas [60, 61] and breast cancer may be concurrent with meningiomas [62]. Immune responses, such as the peritumoral collection of MCs, are increasingly considered to augment tumor growth and metastasis [33, 57, 63].

Two retrospective studies [24, 29] evaluated MCs, by tryptase immunostaining, in a series of meningiomas of various grades. Specimens were divided in two groups of low-grade meningiomas (WHO grade I) and high-grade meningiomas (WHO grades II and III). In the first study [24], 70 cases were analyzed. In the group of low-grade tumors (*n* = 63), MCs were seen in 20/63 cases (31.8 %), with strong diffused immunoreaction in 8/20 mostly next to blood vessels disseminated intratumorally; all psammomatous, secretory, and meningothelial meningiomas were negative for MCs, whereas all fibrous and transitional meningiomas were positive. Interestingly, CT brain images of all MC-positive low-grade meningiomas showed marked peritumoral edema [24].

In the second group (*n* = 7), 6/7 (86 %) tumors were positive for MCs and all presented with peritumoral edema on CT. One anaplastic meningioma was strongly immunopositive, while the rest of the high-grade meningiomas showed focal, but disseminated, positive immunoreaction for tryptase [24]. The second study [29], was conducted on 154 cases, and apart from MCs, it also evaluated the expression of hypoxia-inducible factor-1 (HIF-1), which is a marker of hypoxia found to be correlated with grade and progression of many cancers including glioblastoma [64]. In the group of low-grade meningiomas (*n* = 104), MCs were seen in 42 cases (40.4 %), with strong diffused immunoreaction in 17/42. In the group of high-grade meningiomas (*n* = 50), 45 (90 %) of tumors were positive for MCs, with strong immunoreaction in 6/45 cases. MCs were observed not only next to blood vessels but also within the tumor [29].

Based on the Steinhoff classification of peritumoral edema [65], this study showed a statistically significant association between HIF-1 expression, tryptase expression, and the presence of peritumoral brain edema, as well as between MC accumulation and HIF-1 expression based on meningioma grading [29]. MC mediators such as histamine, serotonin, or VEGF might significantly contribute to the formation of peritumoral edema.

Tirakotai et al. studied secretory meningiomas (grade I), which were found to be infiltrated by a higher number of MCs compared to other types (nonsecretory meningiomas). Higher number of MCs was found mainly in and around the pseudopsammoma bodies of secretory meningiomas [66] Eparil et al. and Tina-Suck et al. investigated 12 and 10 cases, respectively, of chordoid meningiomas (grade II) [67, 68]. Both reported a significant number of MCs in this meningioma variant. The first observed MCs both within the myxoid stroma and the epithelial cell islands, by using toluidine blue and Giemsa stains, in 100 % of cases. MCs were sparsely populated, granulated, single in arrangement, and more frequently seen at the interface regions [67]. The second observed MCs present both in the connective tissue stroma and the epithelial cell islands in all specimens, using positive Periodic Acid Schiff (PAS) and mucicarmine stains [68]. In contrast, another study [30] reported that MCs had poor associations with meningioma tumor grading.

### Mast cells in cystic meningiomas

**Fig. 1:**
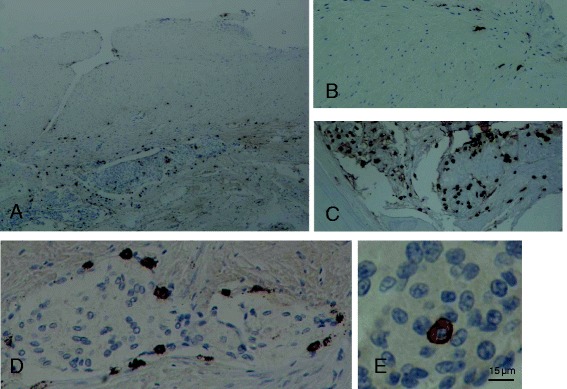
Photomicrographs of tissue samples of dura and bone infiltrated by meningioma of the meningothelial type grade I obtained from a brain lesion following a left-left frontotemporal craniectomy. Mast cells were stained immunohistochemically for the presence of tryptase (*brown color*). **a** Dura showing the upper unaffected part and the lower part infiltrated meningioma cells (*blue*) and accumulated mast cells (*brown*); magnification = ×40. **b** Unaffected dura; magnification = ×100. **c** Bone infiltrated by meningioma cells showing a cluster of mast cells; magnification = ×100. **d** Mast cells surrounding clusters of meningioma cells infiltrating the dura; magnification = ×400. **e** One mast cell surrounded by meningioma cells; this mast cell does not appear to be degranulated. Bar = 15 μm

**Fig. 2:**
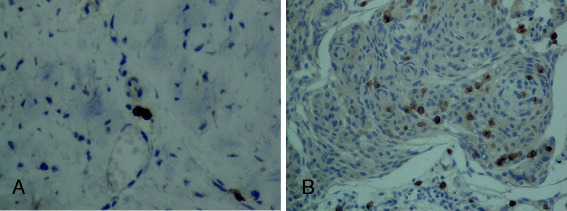
Photomicrographs of tissue samples of dura infiltrated by meningioma of the meningothelial type grade I obtained from a brain lesion following a left-left frontotemporal craniectomy. Mast cells were stained immunohistochemically for the presence of CD117 (*brown color*). **a** Unaffected dura showing two mast cells. **b** Dura infiltrated by meningioma cells showing a number of mast cells (*brown color*). Magnification = ×200

An interesting role of MCs in meningiomas was revealed in a retrospective study investigating microcystic and cystic changes in 397 meningiomas [69]. Such changes were present in about 10 % of these tumors, mainly of the meningothelial type (grade I), and were associated with permeability disturbances and increased number of MCs. Here we show the presence of MCs in dura and bone infiltrated by meningioma of the meningothelial type grade 1 stained for tryptase (Fig. 1) and CD117 (c-kit, the tyrosine kinase surface receptor for stem cell factor) (Fig. 2). It should be noted that these MCs do not appear degranulated. We recently reported that MCs infiltrating pancreatic adenocarcinoma were also increased, but not degranulated in contrast to MCs in acute pancreatitis that were increased in number, but degranulated [70].

### Meningiomas and perivascular edema

Cerebral edema is quite common in intracranial meningiomas. In one study of 68 meningiomas evaluated by computed tomography, 40 % had significant edema [71]. Another review concluded that intracranial meningiomas were associated with brain edema in 50–66 % of cases [72]. In fact, edema has been considered a prognostic factor for meningiomas and metastases, but not gliomas [73]. Moreover, there was a strong correlation between brain edema and shape of tumor margins and signal intensity on magnetic resonance imaging of 51 meningiomas studied [74]. Peritumoral edema may be due to increased expression of vascular endothelial growth factor (VEGF) [75, 76]. In fact, meningiomas can secrete VEGF-A themselves [77]. It is of interest that MCs can secrete large quantities of VEGF especially in response to CRH [78], which can be secreted by MCs [79] and by metastatic cancer cells [80].

Meningiomas are well known to present with severe headaches, even when tumors are small with little edema, and mass effect alone is insufficient to account for this symptom [81, 82]. It is therefore of interest that MCs have been implicated in the pathogenesis of migraines [38] and meningeal MC-neuron interactions are increasingly invoked in the pathophysiology of headaches [83, 84]. Meningeal inflammation was also regarded critical in seizures [85], and there have been a number of cases of patients with seizures due to underlying mastocytosis [46, 86].

The association of MCs with perivascular edema is also important because it may indicate disruption of the BBB, which worsens by stress and contributes to brain metastases [57], multiple sclerosis [87], autism [35, 88], and brain “fog” [89]. It may, therefore, be important to investigate the presence of occult meningiomas in such disorders.

### Treatment options

Brain edema may be reduced with the use of glucocorticoids or anti-angiogenic therapy [77].

Cyclooxygenase (COX) inhibitors have also been considered for the treatment of brain edema [73, 90] especially because COX-2 expression has been reported in astrocyte and microglia in humans [91]. It is of interest that certain natural flavonoids, such as quercetin (3, 5, 7-3΄ 4΄-pentahydroxyflavone), inhibit COX-2 and angiogenesis [92, 93], and its structural analog luteolin (5,7-3′4′-tetrahydroxyflavone) inhibits COX-2 in glioblastoma cells [94]. Quercetin also has antiproliferative activity against human meningiomas [95] and gliomas [96, 97]. Luteolin has synergistic action with COX-2 inhibitors on inducing apoptosis of breast cancer cells [98].

Both quercetin [99] and luteolin inhibit MCs [99–101], especially MC-derived VEGF release. Luteolin also inhibits activation of auto-immune T cells [102, 103].

A methylated luteolin analog (6-methoxyluteolin) was shown to inhibit IgE-stimulated histamine release from human basophilic KU812F [104]. Moreover, we recently showed that tetramethoxyluteolin is a more potent inhibitor of human cultured MCs than luteolin [105].

Luteolin also inhibits activation and proliferation of microglia [106–109], which have been implicated in autism [110]. A luteolin/quercetin-containing formulation in olive fruit oil significantly improved attention and behavior in children with autism [111, 112]. It is interesting that oleocanthal present in olive oil was shown to have COX inhibitory activity [113].

Flavonoids are naturally occurring compounds found mostly in green plants, herbs, and seeds with potent antioxidant, anti-inflammatory, and anticancer properties [114]. Recent reviews have discussed the use of flavonoids in neuropsychiatric [115, 116] and neurodegenerative [117, 118] diseases, especially luteolin in the prevention and/or treatment of brain fog [119].

## Conclusion

There is growing evidence that the accumulation of MCs in meningiomas, mainly in perivascular areas, is associated with the presence of peritumoral edema, especially in high-grade tumors. Furthermore, meningeal MCs may contribute to edema and inflammation involved in headaches and possibly seizures. Finally, meningeal MCs may regulate permeability of the BBB and contribute to the pathogenesis of brain metastases, multiple sclerosis, and autism.
